# Keloid and Hypertrophic Scars Are the Result of Chronic Inflammation in the Reticular Dermis

**DOI:** 10.3390/ijms18030606

**Published:** 2017-03-10

**Authors:** Rei Ogawa

**Affiliations:** Department of Plastic, Reconstructive and Aesthetic Surgery, Nippon Medical School Hospital, Tokyo 113-8603, Japan; r.ogawa@nms.ac.jp; Tel.: +81-3-5814-6208; Fax: +81-3-5685-3076

**Keywords:** keloid, hypertrophic scar, steroid tape, radiation, surgery

## Abstract

Keloids and hypertrophic scars are caused by cutaneous injury and irritation, including trauma, insect bite, burn, surgery, vaccination, skin piercing, acne, folliculitis, chicken pox, and herpes zoster infection. Notably, superficial injuries that do not reach the reticular dermis never cause keloidal and hypertrophic scarring. This suggests that these pathological scars are due to injury to this skin layer and the subsequent aberrant wound healing therein. The latter is characterized by continuous and histologically localized inflammation. As a result, the reticular layer of keloids and hypertrophic scars contains inflammatory cells, increased numbers of fibroblasts, newly formed blood vessels, and collagen deposits. Moreover, proinflammatory factors, such as interleukin (IL)-1α, IL-1β, IL-6, and tumor necrosis factor-α are upregulated in keloid tissues, which suggests that, in patients with keloids, proinflammatory genes in the skin are sensitive to trauma. This may promote chronic inflammation, which in turn may cause the invasive growth of keloids. In addition, the upregulation of proinflammatory factors in pathological scars suggests that, rather than being skin tumors, keloids and hypertrophic scars are inflammatory disorders of skin, specifically inflammatory disorders of the reticular dermis. Various external and internal post-wounding stimuli may promote reticular inflammation. The nature of these stimuli most likely shapes the characteristics, quantity, and course of keloids and hypertrophic scars. Specifically, it is likely that the intensity, frequency, and duration of these stimuli determine how quickly the scars appear, the direction and speed of growth, and the intensity of symptoms. These proinflammatory stimuli include a variety of local, systemic, and genetic factors. These observations together suggest that the clinical differences between keloids and hypertrophic scars merely reflect differences in the intensity, frequency, and duration of the inflammation of the reticular dermis. At present, physicians cannot (or at least find it very difficult to) control systemic and genetic risk factors of keloids and hypertrophic scars. However, they can use a number of treatment modalities that all, interestingly, act by reducing inflammation. They include corticosteroid injection/tape/ointment, radiotherapy, cryotherapy, compression therapy, stabilization therapy, 5-fluorouracil (5-FU) therapy, and surgical methods that reduce skin tension.

## 1. Introduction

Keloids and hypertrophic scars are caused by cutaneous injury and irritation, including trauma, insect bite, burn, surgery, vaccination, skin piercing, acne, folliculitis, chicken pox, and herpes zoster infection. Notably, superficial injuries that do not reach the reticular dermis never cause keloidal and hypertrophic scarring. This suggests that these pathological scars are due to injury to this skin layer and the subsequent aberrant wound healing therein, which is characterized by continuous and histologically localized inflammation. As a result, the reticular layer of keloids and hypertrophic scars contains inflammatory cells, increased numbers of fibroblasts, newly formed blood vessels, and collagen deposits. While keloids and hypertrophic scars are generally first observed around 3 months after injury, this merely reflects the fact that the inflammation of the reticular dermis, which starts immediately after the initial injury, only becomes visible through the epidermis to the naked eye at this time point. Moreover, in the case of surgical wounds, both patients and physicians tend to believe (erroneously) that the sutured wound has matured when the sutures are removed. This is because at this point (i.e., 7–14 days after surgery), the epidermis has regenerated and the wound is closed and dry. However, at this stage, the dermal matrix is still maturing and there is ongoing inflammation in the reticular dermis. If, at this time point, the reticular layer is subjected to external and/or internal stimulation, the inflammation fails to subside and instead becomes increasingly pronounced. This yields pathological scars that eventually become apparent a few months after surgery. The intensity, frequency, and duration of the stimuli determine how quickly the scars appear, the direction and speed of growth, and the intensity of symptoms. The stimuli that influence the characteristics and quantity of keloids and hypertrophic scars include a variety of local, systemic, and genetic factors, as follows. Consequently, it is likely that the clinical differences between keloids and hypertrophic scars merely reflect differences in the intensity, frequency, and duration of the inflammation of the reticular dermis [1,2]. In this paper, we define “keloid” as strongly inflamed pathological scars while “hypertrophic scars” are defined as weakly inflamed pathological scars.

## 2. Local Risk Factors That Increase Dermal Inflammation

Various local factors increase and/or prolong the inflammation of the reticular dermis during wound healing. One is rewounding. For example, the repeated attaching and detaching of earrings to and from ear-pierce holes can induce multiple injuries over a long period of time; this greatly increases the risk of developing earlobe keloids. Another factor is infection. For example, ear-pierce holes are also prone to repeated infections, which can establish unfavorably prolonged inflammation in the reticular dermis. Similarly, being infections, acne and folliculitis also associate with a greater risk of pathological scarring. Secondary wounds caused by scratching chicken pox wounds are also at risk of pathological scarring. In the case of burn wounds, the duration of inflammation is particularly prolonged if the burn wound is large and/or deep. This elevates the risk of pathological scar development. Indeed, it was shown that a burn wound that heals in less than 10 days has a 4% risk of developing into a hypertrophic scar, whereas a burn wound that takes 21 days or more to heal has a 70% or greater risk [3].

In our opinion, of all the many local factors that contribute to pathological scar development, the most important is local mechanical forces. Several lines of evidence support this notion. First, keloids commonly adopt distinct site-specific shapes, namely, the typical butterfly, crab’s claw, and dumbbell shapes on the shoulder, anterior chest, and upper arm, respectively [4]. This, together with our visual analysis using the finite element method [5], suggests that keloid growth is largely determined by the direction of the tension that is applied to the skin around the wound site. For example, the direction of tension on the anterior chest wall is horizontal because of the contraction direction of the pectoralis major muscle. Therefore, keloids on the chest wall always grow horizontally. Moreover, the commonly seen elongated vaccination keloid on the upper arm relates to the fact that people were generally vaccinated during childhood: since the upper arm grows in the direction of the long axis, this places tension on the vaccination wound and the shoulder keloids therefore tend to grow in the direction of the long axis.

Another piece of evidence that indicates the importance of local mechanical forces is that keloids show a marked preference for the locations on the body that are constantly or frequently subjected to tension (such as the anterior chest and scapular regions), whereas they seldom occur in areas where stretching/contraction of the skin is rare (such as the parietal region or anterior lower leg) [6]. This is true even for patients with multiple/large keloids. Moreover, keloids are rare on the upper eyelid. This reflects the fact that eyelid skin is always relaxed, regardless of whether the eyes are open or closed.

In the case of earlobe keloids, mechanical force from the pillow and/or the weight of the earring may also participate in their pathogenesis. These factors, and the weight of the keloid itself, can also increase the risk that the keloid progresses.

## 3. Systemic Risk Factors That Increase Dermal Inflammation

In terms of systemic risk factors, adolescence and pregnancy appear to associate with a greater risk of developing pathological scars [7,8]. It may be that sex hormones such as estrogens and androgens have vasodilatory effects that intensify inflammation, thereby promoting pathological scar development or worsening existing scars. This is supported by our unpublished data, which suggest that the incidence of keloids that are not caused by trauma suddenly increases at around 10 years of age. This implies that the increase in sex steroid levels at the start of adolescence, rather than a higher likelihood of trauma, is responsible for the greater risk of pathological scar development in adolescents.

Our recent study also showed that hypertension associates with the development of severe keloids [9]. That study assessed whether hypertension, a circulating factor, influences local keloid severity. It involved 304 consecutive patients with keloids. Ordinal logistic regression analyses showed that blood pressure associated significantly and positively with both keloid size and number (both *p* < 0.0001). This study provides epidemiological evidence for the possibility that hypertension may aggravate keloids. This association may reflect the fact that hypertension damages blood vessels, thereby increasing inflammation in local tissue.

Another systemic risk factor for pathological scar development and/or progression is inflammation. We experienced the case of an adult woman whose auricular keloids were aggravated during the course of her Castleman disease. Castleman disease is a rare lymphoproliferative disorder that is characterized by the unregulated overproduction of interleukin-6 (IL-6). It leads to systemic lymphadenopathy and constitutional inflammatory symptoms. We found that, when the circulating concentrations of inflammatory cytokines were increased in our patient, her auricular keloids were aggravated. Systemic inflammation is also likely to be responsible for the tendency of patients who undergo reconstructive surgery of extensive burns to develop hypertrophic scars: initial severe burns associate with a cytokine storm that significantly increases the risk of developing keloids and hypertrophic scars for at least 1 year.

## 4. Genetic Risk Factors That Increase Dermal Inflammation

Some keloid and hypertrophic scar patients have a familial history of pathological scarring, which suggests that these scars can be driven by genetic factors. Moreover, there is clinical evidence that patients with darker skin are 15 times more likely than patients with lighter skin to develop pathological scars (primarily keloids) [10]. Moreover, these scars are absent in albinos [11]. The genetic causes of pathological scar development may be single nucleotide polymorphisms (SNPs): a genome-wide association study showed that four SNP loci in three chromosomal regions associate significantly with keloid development in the Japanese population [12]. Moreover, our study showed that one SNP (rs8032158) associates significantly with keloid clinical severity [13]. The rs8032158 SNP is located in intron 5 of the NEDD4 (Neuronal precursor cell-Expressed Developmentally Downregulated 4) gene on chromosome 15. This SNP may contribute to the aberrant cell proliferation that characterizes keloids, although further studies are needed to test this notion. There are probably many other genetic factors that have not yet been identified.

Chromosomal changes also relate to familial keloids. To date, potential keloid-associated loci in Japanese, African-American, and Han Chinese families have been identified on chromosomes 2q23, 7p11 [14], and 10q23.31 [15], respectively, although the responsible genes have not yet been identified.

## 5. Histopathology and Genetic Analysis of Keloid Tissue

Many classical textbooks consider keloids and hypertrophic scars to be distinct types of scar. Clinicians define hypertrophic scars as scars that do not grow beyond the boundaries of the original wound and keloids as scars that spread into the surrounding normal skin. By contrast, pathologists make a histological distinction between keloids and hypertrophic scars on the basis of thick eosinophilic (hyalinizing) collagen bundles called “keloidal collagen”: these are present in keloids but fewer in hypertrophic scars. However, there are many cases where the scar bears the growth and histological features of both hypertrophic scars and keloids. Notably, we have observed that scars that spread into the surrounding normal skin tend to have a large volume of hyalinized collagen and many blood vessels, whereas those that do not grow beyond the boundaries of the original wound generally have a small volume of hyalinized collagen and relatively few blood vessels. Thus, it is possible that hypertrophic scars and keloids are actually manifestations of the same fibroproliferative skin disorder and they just differ in the intensity and duration of inflammation [1,2]. These features may in turn be influenced by local, systemic, and genetic risk factors.

It was reported that proinflammatory factors such as IL-1α, IL-1β, IL-6, and tumor necrosis factor-α are upregulated in keloid tissues [16,17]. As a result, Dong et al. [16] speculated that the proinflammatory genes in the skin of patients with keloids are sensitive to trauma, namely, that they tend to be more readily upregulated on injury to the dermis and more likely to sustain this upregulation than the same genes in other people. This results in chronic inflammation that may also explain the invasive growth of keloids. In addition, the upregulation of proinflammatory factors in keloids suggests that, instead of being skin tumors, keloids and hypertrophic scars are inflammatory disorders of skin, specifically inflammatory disorders of the reticular dermis.

## 6. Treatment of Keloids and Hypertrophic Scars

At present, physicians cannot (or at least find it very difficult to) control systemic and genetic factors. However, they can use a number of treatment modalities that all, interestingly, act by reducing inflammation. They include corticosteroid injection/tape/ointment, radiotherapy, cryotherapy, compression therapy, stabilization therapy, 5-fluorouracil (5-FU) therapy, and surgical methods that reduce tension.

### 6.1. Surgery

Surgical methods that can reduce tension on the edges of the wound will help to decrease the inflammation of the skin [18,19]. To reduce the risk of recurrence of pathological scars, it is advisable to use particular surgical techniques, namely, subcutaneous/fascial tensile reduction sutures, z-plasties, and local flap transfer. However, typical keloids, which have strong levels of inflammation, cannot be treated by surgery alone. For these cases, multimodal therapy, such as surgery followed by radiation therapy and/or steroid administration, may be most suitable.

In general, dermal sutures do not effectively reduce the tension on the dermis: to achieve this, we must access much deeper structures, namely, the superficial and deep fascia, and suture them. This type of suturing will elevate the wound edges smoothly while placing minimal tension on the dermis. In other words, the wound edges naturally attach to each other. Only then should dermal and superficial sutures be used. It is very important to realize that dermal sutures on their own cannot reduce the tension on the dermis: this concept is the key to preventing the formation of pathological scars after surgery.

**Figure 1:**
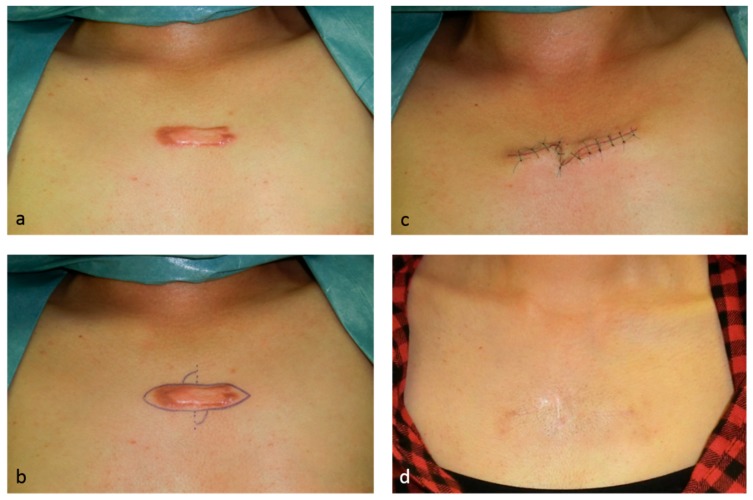
This 24-year-old female had a minor acne keloid on her chest that was treated by surgery, namely, total excision and z-plasty, plus postoperative taping fixation. Since the keloid was small, it could be removed totally and the tension was released by the z-plasty. Immediately after removing the sutures on day 10, silicone tape fixation was started to stabilize the wound. Eighteen months after surgery, recurrence was not observed. (**a**) Preoperative view; (**b**) design of the z-plasty; (**c**) immediately after surgery; and (**d**) 18 months after the operation.

Zig-zag sutures, including z-plasties, are good for releasing the tension on scars (Figure 1). A major benefit of z-plasties is that segmented scars mature faster than long linear scars. In particular, if a scar crosses a joint, zig-zag incision and suturing significantly reduces the risk of pathological scar development.

Various local flaps are also useful for releasing scar contractures. Moreover, because local flaps expand naturally after surgery, they are not prone to postsurgical contractures. By contrast, skin grafts do not expand, which means that skin grafting tends to generate secondary contractures that result in circular pathological scars around the grafted skin. Thus, flap surgery is better for keloids.

### 6.2. Radiotherapy

**Figure 2:**
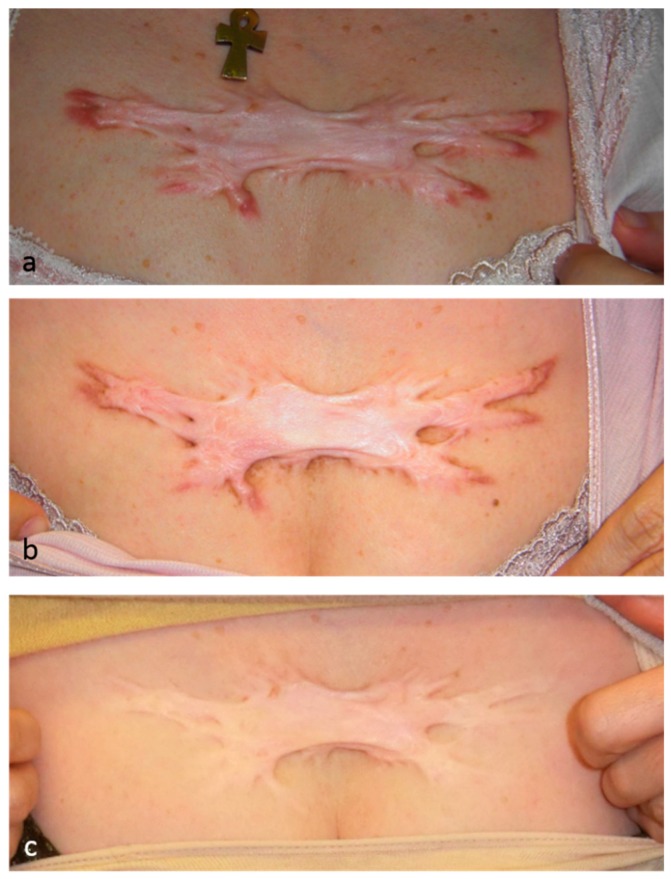
This 51-year-old female had a minor chest wall keloid that was treated by high-dose-rate superficial brachytherapy. A total of 25 Gy was administered in five fractions over 5 days. The inflammation resolved completely. After 1 year of treatment, both the subjective and objective symptoms had improved dramatically. (**a**) Pretreatment view; (**b**) 6 months post-treatment; and (**c**) 4 years post-treatment.

Several studies show that radiotherapy effectively prevents and treats keloids [20,21]. While it is reported that radiotherapy primarily acts by suppressing fibroblast activity, it should be noted that endothelial cells are more sensitive to radiation than fibroblasts. It is thus reasonable to consider that radiotherapy largely acts by suppressing angiogenesis: this in turn prevents the formation of dysfunctional blood vessels and decreases inflammation, thereby suppressing keloid development. This notion is supported by the fact that, when we subject patients with severe keloids to radiation monotherapy, the color improves almost immediately, after which the scar becomes progressively flatter (Figure 2). This pattern is consistent with the idea that radiotherapy first decreases blood vessel numbers, which then reduces inflammation, which finally dampens fibroblast activity. These observations suggest that fibroblasts largely act in pathological scar development and progression by receiving and proliferating in response to inflammatory signals derived from blood vessels [2].

### 6.3. Corticosteroid Administration

Corticosteroid injections, ointments, and tapes/plasters effectively treat keloids and hypertrophic scars. In addition to their direct anti-inflammatory effect, we believe that the steroids also act by inducing vasoconstriction. This is supported by the fact that corticosteroid administration causes keloids to whiten: this suggests that the blood flow in the scar has been decreased by vasoconstriction. The vasoconstrictor effects of topical steroids seem to be mediated by their binding to classical glucocorticoid receptors, rather than by nonspecific pharmacological mechanisms. Moreover, corticosteroid administration rapidly reduces the itching and pain associated with keloids, possibly because the vasoconstriction prevents the perivascular delivery of the inflammatory factors that elicit these symptoms.

**Figure 3:**
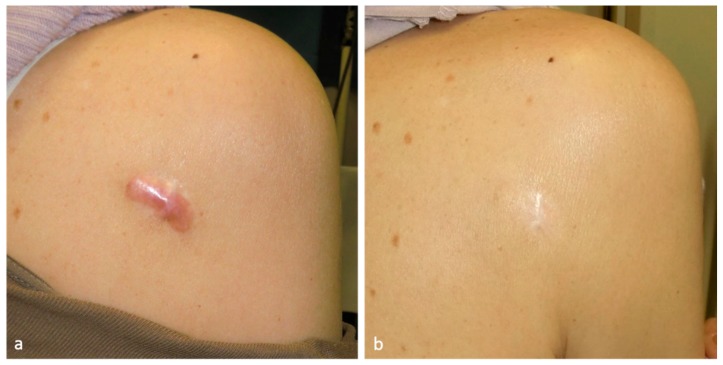
This 75-year-old female had a mild right scapular keloid that was treated by deprodone propionate tape (Eclar^®^ plaster). The tape was placed on the keloid 24 h a day and was changed daily. The inflammation resolved completely. After 19 months of treatment, both the subjective and objective symptoms of the patient had improved dramatically. (**a**) Pretreatment view; and (**b**) after 19 months of treatment.

Along with steroid injection, steroid tape has been used to decrease the inflammation of keloids and hypertrophic scars [19]. This practice is particularly common in Japan and several other countries (Figure 3). Flurandrenolide tape (Cordran^®^ tape), fludroxycortide tape (Drenison^®^ tape), betamethasone patch (Betaflam^®^), and deprodone propionate plaster (Eclar^®^ plaster) are available worldwide. These steroid tapes/plasters should be changed every 24–48 h and should be cut so that they just cover the wound, with minimal attachment (if any) to healthy skin (unpublished data). Since these tapes differ in terms of the strength of the steroid, the most appropriate tape/plaster should be selected on a case-by-case basis.

### 6.4. Compression Therapy

Compression therapy reduces both the objective and subjective symptoms of keloids and hypertrophic scars. We speculate that it acts by occluding the blood vessels in the scar, thereby inhibiting the inflammatory signals coming from the blood vessels. An additional mechanism may be that it stabilizes the wound, thereby decreasing inflammation.

### 6.5. Stabilization Therapy

Since stretching wounds can evoke inflammation of the reticular dermis, wounds should be stabilized. Thus, prolonged external mechanical support using tapes, sheets, and/or garments is recommended for scar prevention. This is supported by our study, which showed that silicone gel sheets reduce the tension on the wound site.

Silicone tape is better than paper tape as it prevents the epidermal injury caused by repeated taping. Moreover, silicone tape keeps the scar surface moist. These tapes can be kept in place until they detach naturally. The patient does not need to change the tape after taking a bath/shower. In our experience, patients generally keep silicone tape in place for about 1–2 weeks. The exception is in summer: perspiration can reduce tape adherence. However, if a patient has a clear history of pathological scars, then stabilization tapes should be exchanged for steroid plaster/tape.

### 6.6. Laser Therapy

**Figure 4:**
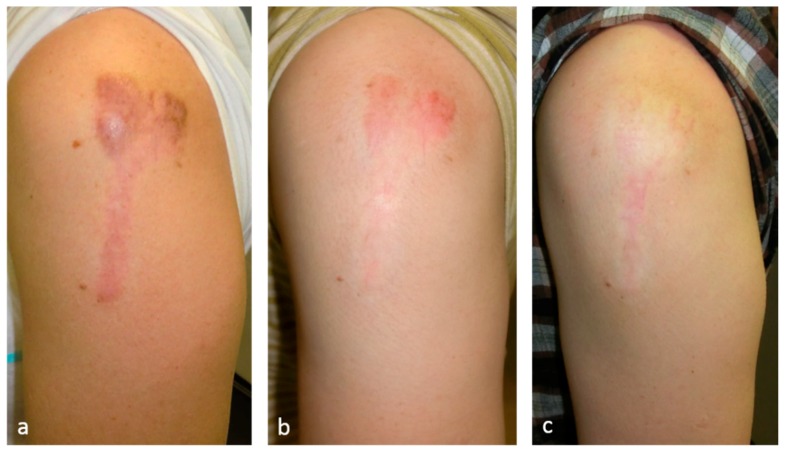
This 60-year-old female had a mild keloid that was treated by 1064 nm long-pulsed Nd:YAG laser. Long-pulsed 1064 nm Nd:YAG laser was used at the following settings: 5 mm spot diameter, 75 J/cm^2^, 25 msec, and 2 Hz. After 1 year of treatment, the scar had almost disappeared. The known target of long-pulsed Nd:YAG lasers is the blood vessels. Thus, we speculate that laser therapy largely treats pathological scars by decreasing blood vessel numbers, thereby inhibiting the inflammatory signals coming from the blood vessels. (**a**) Pretreatment view; (**b**) after 10 months of treatment; and (**c**) after 16 months of treatment.

Pulsed dye laser [22] or long-pulsed Nd:YAG laser [23] therapy reduces the objective and subjective symptoms of keloids and hypertrophic scars (Figure 4). The known target of these lasers is the blood vessels. Thus, we speculate that laser therapy largely treats pathological scars by decreasing blood vessel numbers, thereby inhibiting the inflammatory signals arriving from the blood vessels.

### 6.7. 5-FU Therapy

5-FU treatment has been used successfully to treat keloids. Moreover, several papers show that intralesional 5-FU treatment after surgery prevents recurrence [24]. One of the mechanisms by which 5-FU acts is angiogenesis blockade. Thus, 5-FU may decrease the inflammation in the scar.

### 6.8. Cryotherapy

The mechanism by which cryotherapy reduces keloids is interesting. While hypertrophic scars and keloids occur on burned skin areas, they never develop on frostbitten areas. It appears that, while burning and frostbite both induce apparent tissue necrosis, they induce the secretion of quite different proinflammatory mediators; the response to these inflammatory signals by the fibroblasts may also differ.

Cryotherapy has been used to treat keloids either as a monotherapy or in combination with intralesional triamcinolon injection [25]. Cryotherapy delivery methods include contact, sprays, or intralesional needles.

### 6.9. Other Therapies

In recent years, fat grafting/lipofilling [26], stem cell therapy [27], and electrochemotherapy [28] have been used to treat burn scars, hypertrophic scars, and scar contractures. The effectiveness of these techniques remains unclear. Large-scale clinical trials on these techniques are expected. Moreover, it is likely that, in the next few years, a number of drugs that target specific molecules and thereby improve or prevent pathological scarring will be tested in clinical trials. These drugs may target molecules that participate in inflammation and angiogenesis in the reticular dermis. In particular, drugs that target mechanosensors may be highly effective given that pathological scars are sensitive to mechanical forces [29].

## 7. Conclusions

In recent years, our understanding of the pathogenic mechanisms that drive keloid and hypertrophic scar development and progression has grown rapidly. These pathological scars are essentially the result of chronic inflammation of the injured reticular dermis. Therefore, treatment strategies against these scars should focus on preventing or dampening inflammation. Since these scars are not tumors, the therapeutic target should be the blood vessels, the endothelial cells, or the perivascular cells, rather than the fibroblasts. This is supported by our clinical experiences, which show that many of the methods that effectively treat pathological scars act by targeting the blood vessels.
